# Preparation of Thermochromic Microcapsules of Bisphenol A and Crystal Violet Lactone and Their Effect on Coating Properties

**DOI:** 10.3390/polym14071393

**Published:** 2022-03-29

**Authors:** Wenting Zhao, Xiaoxing Yan

**Affiliations:** 1 Co-Innovation Center of Efficient Processing and Utilization of Forest Resources, Nanjing Forestry University, Nanjing 210037, China; zhaowenting@njfu.edu.cn; 2 College of Furnishings and Industrial Design, Nanjing Forestry University, Nanjing 210037, China

**Keywords:** thermochromic microcapsules, paint film property, microcapsule content

## Abstract

Thermochromic microcapsules were prepared with a thermochromic compound as core material and urea formaldehyde as wall material. The molar ratio of urea and formaldehyde, the mass ratio of core material to wall material, the concentration of emulsifier, and rotating speed were selected to make a four-level and three-factor L_9_(3^4^) orthogonal test. It was found that the molar ratio of urea and formaldehyde had the greatest influence on the coating rate of microcapsules. The effects of molar ratio of urea and formaldehyde on the discoloration temperature and coating rate of microcapsules were studied. When the molar ratio of urea to formaldehyde was 1:1.6, the core material: wall material ratio was 4:7, the concentration of emulsifier was 5.0%, and the rotating speed was 1600 rpm, the performance of the microcapsules was the best. When the microcapsule content was 20.0%, the color difference of the paint film was the largest, the gloss and hardness of the paint film decreased with increasing microcapsule content, and the impact resistance of the paint film first increased and then decreased with increasing microcapsule content. The adhesion of the paint film remained unchanged, and was grade 1. When the microcapsule content was 20.0%, the performance of the paint film was at its best. This provides a basis for the application of thermochromic coatings.

## 1. Introduction

As a renewable and recyclable green material, wood plays an important role in people’s production activities [1,2,3,4]. The easy-to-process nature has led wood to be widely used in structures, doors and windows, furniture, decorations, and so on [5,6,7,8]. Lignocellulose and hemicellulose are easily degraded into glucose, water and carbon dioxide, making the wood susceptible to decay, moths, and mildew [9,10,11]. Coatings can effectively solve these problems and protect the wood [12].

A microcapsule is a kind of microparticle with a shell–core structure composed of core material and wall material [13]. A thermochromic microcapsule is a microcapsule coated with a thermochromic compound as the core material. When the temperature reaches the thermochromic temperature, the microcapsule changes color [14,15]. Pedaballi et al. [16] measured the sedimentation, rheological behavior, and dispersion properties of thermochromic microcapsules in toluene and coating resin. It was found that paint films containing dispersed microcapsules showed better surface flatness and small pores in the microstructure. The paint film with thermochromic microcapsules responded to temperature changes. Ma et al. [17] prepared temperature-reversible thermochromic cement by adding reversible thermochromic microcapsules into white Portland cement. It was found that when blue, green, or red reversible thermochromic microcapsules with a discoloration temperature of 30 °C were added, these reversible thermochromic microcapsules could reversibly change from colored to white, and the conversion temperatures were about 42 °C and 58 °C. Du et al. [18] compared the effects of thermochromic microcapsules on Rtheological and aging behaviors of base and styrene–butadiene–styrene copolymer (SBS)-modified asphalt binders. Thermochromic microcapsules improved fatigue resistances in both base and SBS-modified asphalt binders. Thermochromic microcapsules had little effect on the rutting and cracking properties of SBS-modified asphalt binder. Ma et al. [19] combined thermochromic materials with optimized disperse cationic dyes and designed a new type of thermochromic microcapsule to expand the color range of thermochromic materials. Yang et al. [20] prepared cholesteric liquid crystals (CLC) microcapsules containing cellulose nanocrystals (CNC) and melamine–formaldehyde (MF) resin hybrid shell by means of in situ polymerization with CNC-stabilized Pickering emulsion as template. A thermochromic coating was prepared by mixing CLC microcapsules with a curable adhesive. It was found that the thermochromic paint film could adjust the color within a temperature range of 12 to 42 °C and throughout the wavelength range of visible light. In addition, the obtained thermochromic paint film showed a relatively high reflectivity of up to 30–40%.

There are various preparation methods for obtaining thermochromic microcapsules, such as in situ polymerization, interfacial polymerization, complex coacervation, etc. Because of its simple preparation process, small number of operation procedures, and the fact that the raw materials of the microcapsules are simple and easily available, in situ polymerization has become a common method for preparing microcapsules. In situ polymerization refers to a method in which the monomer (wall material) and the catalyst are located inside the core material or in the microencapsulated medium. The formation conditions require that the monomer is soluble and the polymer is insoluble. In this way, the generated polymer can be deposited on the surface of the capsule core to form microcapsules.

In this paper, the microcapsules were prepared by means of in situ polymerization. The thermochromic compound was coated as the core material of the microcapsules, and the thermochromic microcapsules were applied to coatings. The wall material of the microcapsules consisted of urea-formaldehyde resin, and was hard, tough, highly transparent, and exhibited chemical resistance. The core material was a thermochromic compound of bisphenol A, tetradecanol, and crystal violet lactone with high sensitivity and low color changing temperature, and the optimal process for the preparation of microcapsules was explored. The thermochromic microcapsules were applied to the paint film in order to verify the discoloration effect of the thermochromic paint film, thus broadening the application range of the coatings and increasing their aesthetic value.

## 2. Materials and Methods

### 2.1. Experimental Materials

The experimental drugs used in this paper are shown in Table 1.

### 2.2. Experimental Method

The molar ratio of urea to formaldehyde, the mass ratio of core material to wall material, emulsifier concentration and rotating speed were selected on the basis of the literature and previous experiments to make a four-level and three-factor L_9_(3^4^) orthogonal test table, as shown in Table 2 and Table 3.

#### 2.2.1. Preparation of Thermochromic Microcapsules

Preparation of core material (thermochromic compound): Tetradecanol was heated to a molten state, a certain mass was accurately weighed and put into a beaker, and then bisphenol A and crystal violet lactone of corresponding mass were added. The ratio of leuco: chromogenic agent: solvent was 1:3:60. The agitator was put into the water bath and heated to 50 °C for stirring. After stirring evenly, the water bath was slowly heated to 90 °C and stirred at 400 rpm for 1.5 h. It was found that the solution became clear and transparent. After cooling to room temperature, the resulting core material with discoloration temperature of 25 °C had been produced.

The emulsifier (gum acacia), distilled water and thermochromic compound of corresponding quality in the beaker were added, and the water bath pot was heated to 50 °C and stirred slowly until the compound was completely dissolved. Then the temperature was raised to 65 °C and stirred at the corresponding speed for 20 min. The stirred solution was put into the ultrasonic emulsifying machine for 5 min, so that the emulsifier was evenly wrapped on the outer surface of the core material.

Preparation of wall material (urea formaldehyde resin): According to Table 4, urea and formaldehyde of corresponding quality were weighed and stirred in a water bath at room temperature until they were completely dissolved. The triethanolamine solution was absorbed and slowly added to the urea formaldehyde solution; the pH value of the solution was adjusted to about 8.5, and then the beaker was sealed, the water bath was heated to 70 °C, and it was stirred at a speed of 300 rpm for 1 h.

Preparation of microcapsules: The core material emulsion obtained after ultrasonication was put into a 35 °C water bath pan, stirring slowly. The separating funnel was used to dissolve the wall material and added it into the core material solution drop by drop. Then, the rotating speed of the water bath was adjusted to 500 rpm, the mass ratio of silica to sodium chloride was 8%, citric acid aqueous solution was added, the pH value of the solution was reduced to about 2.5, and the reaction took 1 h. After the reaction was completed, the temperature was increased to 68 °C, the rotating speed was reduced to 250 rpm, and the reaction was continued for 30 min to obtain the microcapsule solution. It was filtered by vacuum pump, and the blue powder obtained after drying in an oven at 35 °C comprised the color-changing microcapsules.

#### 2.2.2. Single-Factor Experiment

Table 5 presents the list of materials for the single-factor experiment. The molar ratio of urea to formaldehyde was set as 1:2, 1:4, 1:5, 1:6 and 1:8. According to the orthogonal test results, other factors were determined as follows: core material: wall material ratio was 4:7, emulsifier concentration was 5.0%, and rotational speed was 1600 rpm.

#### 2.2.3. Preparation of Thermochromic Paint Film

**Figure 1:**
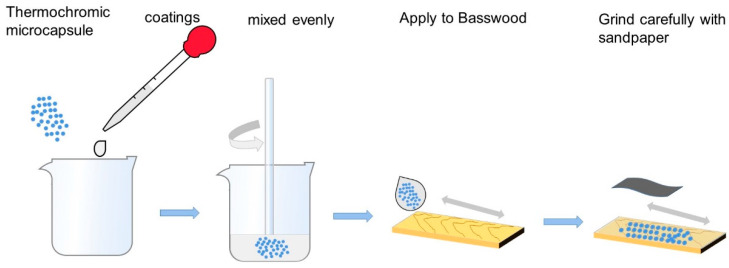
Preparation process of paint film.

Figure 1 shows the coating process employed to prepare the paint film. Table 6 presents a list of the experimental ingredients of paint film. Firstly, the microcapsules and coatings of corresponding quality were weighed and stirred evenly. Then, the microcapsules and coatings were evenly coated on the surface of Basswood with a paint film preparer, dried at room temperature for 1 h, finely polished with 1000 mesh sandpaper [21], and the floating powder was wiped off to make the surface of paint film smooth and flat. The paint film thickness was about 60 µm [22,23].

### 2.3. Testing and Characterization

Table 7 present the test instruments used in this experiment. 

#### 2.3.1. Testing and Characterization of Microcapsules

Microcapsule morphology analysis: The microcapsules were placed on a clean slide and covered with a cover slide; the slide containing the microcapsule was placed on the table and fixed, the testing software was opened, the light source and magnification were adjusted, and the slide was observed with the biological microscope. The microcapsules were evenly pasted on double-sided adhesive tape, and then sample preparation was carried out by means of a gold spraying technique. After vacuumizing, it was observed using a scanning electron microscope.

Microcapsule coating rate test: A certain weight of microcapsules was weighed and recorded as *m*_1_. A certain amount of anhydrous ethanol was added. After mixing evenly, it was sealed with membrane and put into an oven at about 40 °C. It was fully stirred with a glass rod every 15 min. After mixing, it was put into the oven again. After 2 h, the remaining substances were pumped and filtered, dried, and weighed, and the result was recorded as *m*_2_. The coating rate (*P*) of the microcapsules was calculated according to the following formula [24]:

*P* = (*m*_1_ − *m*_2_)/*m*_1_ × 100% (1)

Discoloration temperature of thermochromic microcapsules: Microcapsules of equivalent quality were taken out and placed in the oven. The oven temperature was set to 40 °C, and was increased by 2 °C at each time step. After reaching the specified temperature, it was kept at that temperature for 10 min to fully heat the microcapsules. During the heating process of the oven, the color change of the microcapsules was observed, and the color change temperature of the microcapsules was recorded.

Solvent resistance: Three groups of 0.02 g microcapsules and thermochromic compound were weighed and placed in six test tubes. Then, 2.0 g acetone, 2.0 g ethyl acetate, and 2.0 g xylene solution were added to each test tube, sealed with a rubber band and fresh-keeping film, and placed on the test tube rack. After 24 h, the color change and dissolution of microcapsules and thermochromic compounds was observed.

Acid and alkali resistance: Three groups of 0.02 g microcapsules and thermochromic compound were weighed and placed in six test tubes. Then, 2.0 g of 0.1 mol/L acetic acid solution, 2.0 g of 0.1 mol/L hydrochloric acid solution, and 2.0 g of 0.1 mol/L triethanolamine solution were added to each test tube. They were sealed with rubber band and fresh-keeping film and placed on the test tube rack. After 24 h, the color change and dissolution of microcapsules and thermochromic compounds were observed.

The thermal stability of the microcapsules was measured by thermogravimetric analyzer, and the temperature range was 35 °C–500 °C.

#### 2.3.2. Testing and Characterization of Paint Film

The color difference of the paint film was tested with a portable colorimeter. The portable color difference meter was turned on and calibrated. After that, the test sample was tested, and the values of *L*, *a* and *b* were recorded. The *L*, *a*, and *b* values represent the color space coordinates of the color at that location, where the *L* value represents the lightness value. The value of *a* represents the red–green value. When the value of *a* is positive, the color is reddish; when the value of *a* is negative, the color is greenish. *b* represents the yellow–blue value. When the value of *b* is positive, the color is yellowish; when the value of *b* is negative, the color is blueish. The color difference (Δ*E*) between two points was calculated according to the following formula [25]:

(2) $$\Delta E=\sqrt{{\left(L_{1}-L_{2}\right)}^{2}+{\left(a_{1}-a_{2}\right)}^{2}+{\left(b_{1}-b_{2}\right)}^{2}}$$

**Figure 2:**
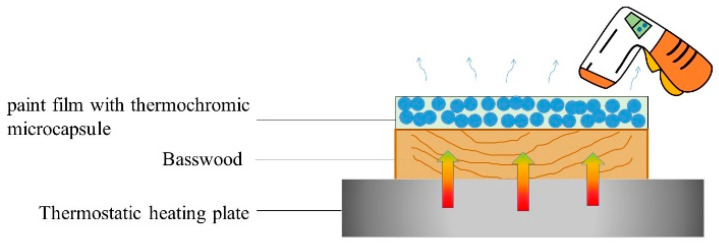
Temperature test diagram of paint film.

Figure 2 presents a diagram of the temperature test for the paint film. The heating plate was used to test the discoloration temperature of the paint film. While heating the sample with the heating plate, the temperature change on the film surface was measured with a thermometer. The temperature range was set to 50 °C–80 °C, and the chromaticity value was tested once every 5 °C. After heating to the target temperature, this temperature was maintained for 10 min to fully heat the paint film, so that the heat entered the microcapsule inside the paint film, and the chromaticity value of the paint film was measured after 10 min. The color difference was calculated to explore the discoloration temperature of the paint film.

The gloss of the paint film was tested according to GB/T 4893.6-2013 [26]. The film gloss was recorded at 20°, 60° and 85°. The hardness of the paint film was tested according to GB/T 6739–2006 [27]. The pencil was inserted into the pencil hardness tester and rolled forward. If the paint film was not damaged, the maximum hardness of the pencil was taken as the hardness of the paint film. The adhesive force of the paint film was measured according to GB/T 4893.4–2013 [28]. The paint film scriber was used to cut the paint film, and then adhesive tape was attached to the paint film and torn off, and the adhesive force of the paint film was judged according to the peeling area of the paint film. The impact resistance of the paint film was determined according to GB/T 1732-1993 [29]. A QCJ−50 coating impact tester was used to test the impact resistance of the paint film. The maximum height of the paint film without cracking was taken as the impact strength.

The aging test was carried out in an aging resistance test chamber according to GB/T 1865-2009 [30]. The xenon lamp irradiance was set to 50 W/m^2^, and paint film coated with thermochromic microcapsules was placed in the UV lamp box until the paint film exhibited no discoloration properties [31,32].

## 3. Results and Discussion

### 3.1. Morphological Analysis of Microcapsules for the Orthogonal Test

**Figure 3:**
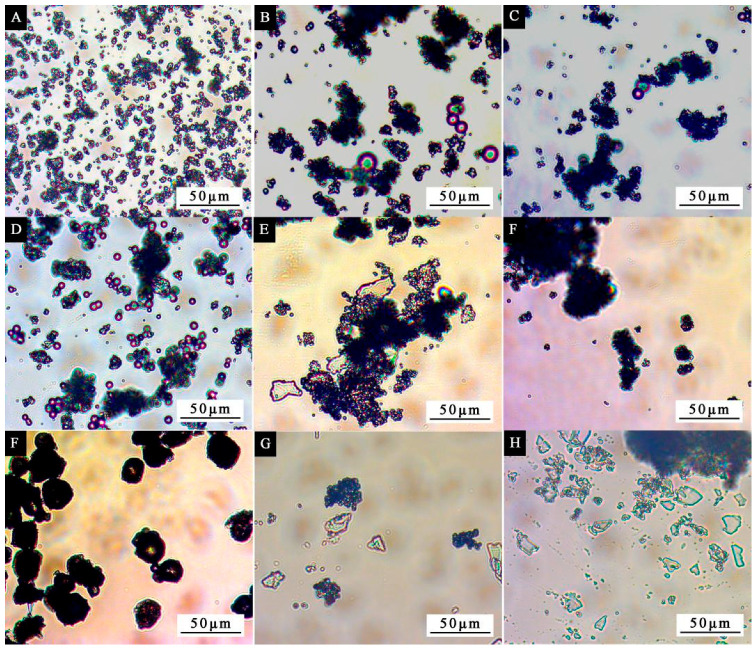
OM of microcapsule in orthogonal test. (**A**–**H**) Samples 1–9.

Figure 3 shows the optical microscope image of the microcapsule in the orthogonal test. As can be seen from Figure 3, the lower the core–wall ratio of the microcapsules, the rounder the shape and the more uniform the dispersion of the microcapsules. When increasing the core–wall ratio of microcapsules, the agglomeration phenomenon of the microcapsules became more and more serious, and the particle size of the microcapsules became more and more uneven. When increasing the molar ratio of urea to formaldehyde, the number of transparent crystals in the image gradually increased, and these transparent crystals were uncoated core materials. The higher the molar ratio of urea and formaldehyde, the more dihydroxymethylurea is generated. The higher the degree of crosslinking, the denser the wall material of the microcapsules. However, excessive participation of formaldehyde in the reaction produces a large number of hydrophilic hydroxymethyl groups during the polycondensation process, resulting in incomplete polycondensation and failure to completely cover the core material during curing, thus reducing the coating rate of the microcapsules.

**Figure 4:**
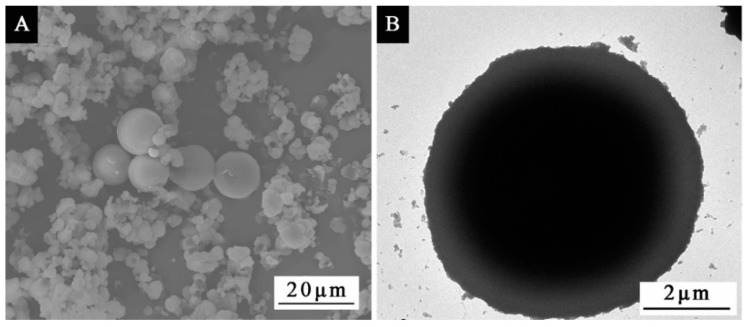
SEM (**A**) and (**B**) TEM of thermochromic microcapsules.

Figure 4 shows the scanning electron microscope (SEM) and transmission electron microscope (TEM) images of orthogonal experimental sample 2. From the SEM images, it can be seen that the microcapsules of sample 2 were round in shape and possessed a relatively uniform particle size distribution. From the TEM images, it can be seen that the outer layer of microcapsules was light in color, and the inner layer was dark in color, and the light layer was the wall polymer outside the microcapsules [33]. From the SEM and TEM images, it can be seen that the microcapsules were shell–core structures.

**Figure 5:**
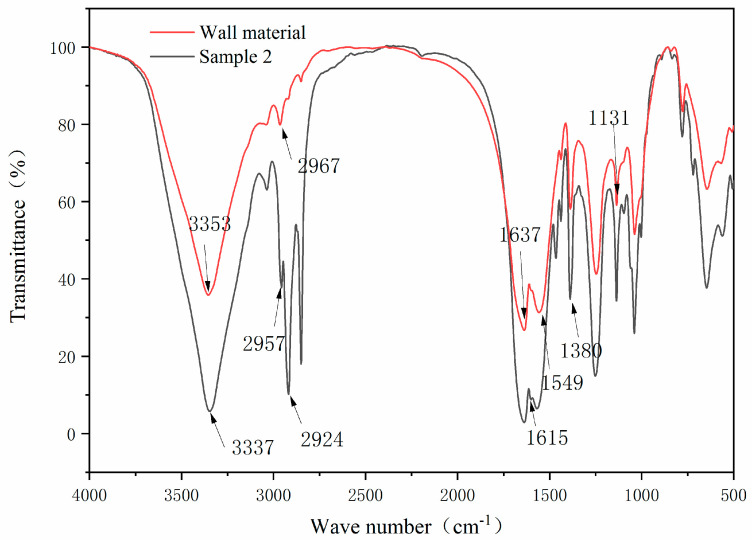
FTIR spectra of urea formaldehyde resin and thermochromic microcapsules.

Figure 5 presents the infrared spectrum of the wall material in comparison with that of sample 2. It can be seen from the FTIR of the wall material that the stretching vibration peaks of N-H bond and O-H bond were superimposed at 3353 cm^−1^, the asymmetric stretching vibration of -CH_2_- was at 2957 cm^−1^, and the carbonyl stretching vibration peak of secondary acyl was at 1637 cm^−1^. The peak at 1549 cm^−1^ was caused by the bending vibration of the N-H bond of -(C=O)-NH- and the stretching vibration of the C-N bond. The characteristic absorption peak of CH_3_O was at 1131 cm^−1^ [34]. These peaks indicated the successful preparation of urea formaldehyde resin.

In the infrared spectrum of microcapsules, the characteristic peak of the core material also appeared, the ester carbonyl C=O absorption peak of the non-lactone ring structure appeared at 1615 cm^−1^, and the peak of 1368 cm^−1^ corresponded to the symmetrical stretching absorption peak of carboxylate, which proved that the lactone ring in the molecule was open and formed a conjugated chromogenic structure. The peaks at 2955 cm^−1^ and 2917 cm^−1^ belonged to the asymmetric stretching vibration and symmetric stretching vibration of C-H in tetradecanol and methyl in crystal violet lactone [35]. These characteristic peaks indicated the successful preparation of thermochromic microcapsules.

### 3.2. Orthogonal Experimental Analysis

Table 8 presents the visual analysis table for the orthogonal test. It can be seen from Table 8 that the coating rate of sample 5 was the highest, at 54.0 ± 1.35%. When the molar ratio of urea to formaldehyde was 1:1.5, the core material-to-wall material ratio was 4:7, the concentration of emulsifier was 5.0%, and the rotating speed was 1600 rpm, the coating rate of the microcapsules reached its highest value. On the basis of the analysis of variance table (Table 9), it can be seen that the molar ratio of urea and formaldehyde had the greatest impact on the coating rate of microcapsules, followed by emulsifier concentration, speed, and finally the ratio core material: wall material. Therefore, the single-factor optimization experiment was designed with the molar ratio of urea to formaldehyde as the variable.

It can be seen from Table 10 that the reagent resistance and acid–base resistance of the thermochromic compound were poor. After 24 h, the thermochromic compound dissolved, and the original blue compound became colorless. Compared with the thermochromic compound, the solvent resistance and acid–base resistance of the microcapsule were greatly improved. After 24 h, the color of the microcapsule was still blue, and without dissolution; this showed that the microcapsule technology had a protective effect on the thermochromic compound, and urea formaldehyde resin is able to significantly improve the solvent resistance and acid–base resistance of the thermochromic compound.

### 3.3. Morphological Analysis of Microcapsules for the Single Factor Test

**Figure 6:**
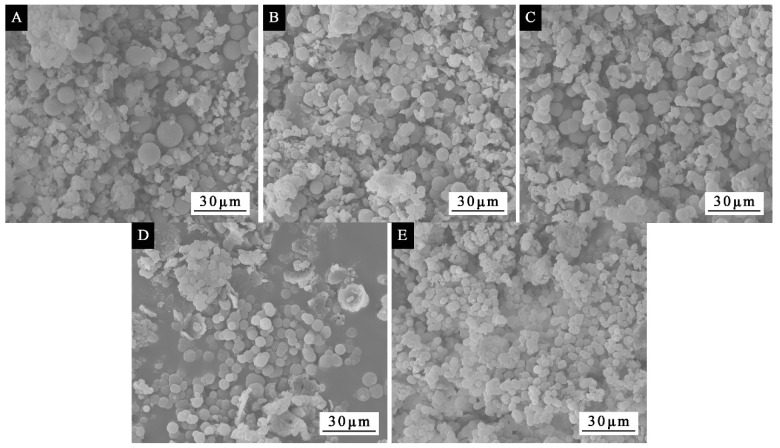
SEM of single-factor experimental microcapsules: molar ratio of urea to formaldehyde (**A**) 1:1.2, (**B**) 1:1.4, (**C**) 1:1.5, (**D**) 1:1.6, (**E**) 1:1.8.

Figure 6 showed the SEM of microcapsules with different molar ratios. It can be seen from the figure that the particle size of the microcapsules gradually became uniform and decreased with increasing molar ratio. The microcapsules with molar ratios of 1:1.5 and 1:1.6 showed a uniform circular appearance without serious agglomeration. The particle size was uniform, and the diameter was about 5 μm. The greater the amount of formaldehyde involved in the reaction, the more dimethylol urea was produced, the higher the crosslinking degree of the microcapsules, and the tighter the surface of the microcapsules.

Table 11 presents the yield of the single-factor microcapsules. It can be seen from the table that the output of microcapsules with different molar ratio first increases and then decreases with increasing molar ratio. When the molar ratio was 1:1.4, the output reached its highest value, which was 15.91 ± 0.39 g, and when the molar ratio was 1:1.8, the output was at its lowest, which was 12.63 ± 0.31 g.

Table 12 shows the coating rate of single-factor microcapsules. It can be seen that with increasing molar ratio, the coating rate of the microcapsules gradually decreased. When the molar ratio was 1:1.2, the coating rate of microcapsules reached its highest value, which was 70.0 ± 1.75%, and when the molar ratio was 1:1.8, the coating rate of microcapsules reached its lowest value, which was 53.3 ± 1.33%. This is because too much formaldehyde would result in the inclusion of a large number of unreacted hydroxymethyl hydrophilic groups in the polycondensation product, causing the polycondensation to be incomplete, resulting in a low microcapsule encapsulation rate.

Table 13 shows the thermochromic temperature of microcapsules with different core–wall ratio. It can be seen from this that with increasing molar ratio, the discoloration temperature of microcapsules tended to first rise, then decrease, and then rise again. When the molar ratio of urea to formaldehyde was 1:1.6, the thermochromic temperature reached its lowest value, at 65.00 ± 1.62 °C, while the highest value of 95.00 ± 2.37 °C was obtained when the molar ratio was 1:1.8.

**Figure 7:**
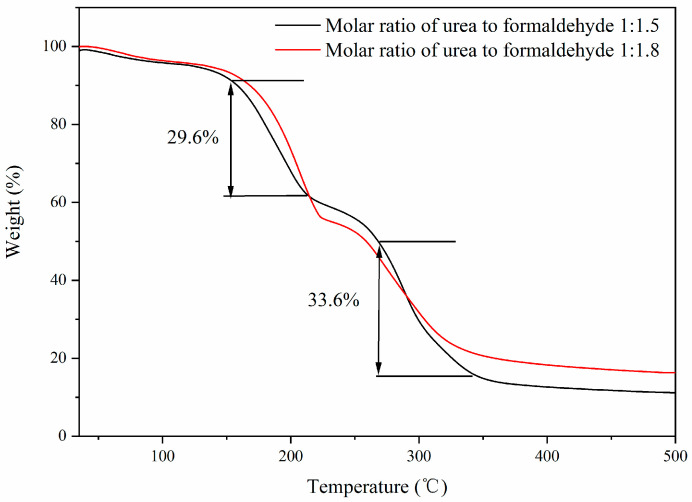
TGA of thermochromic microcapsules with different molar ratios.

Figure 7 presents the TGA of the microcapsules. From the TGA of the microcapsules, it can be seen that the microcapsules had two thermal weight loss stages. When the temperature was about 150 °C, the microcapsules underwent a first thermal decomposition, with a thermal weight loss rate of 29.6%. This is because the wall material of the microcapsule was not sufficiently covered, causing some core materials to dissolve. When the temperature was about 270 °C, the microcapsules underwent a second thermal decomposition, with a thermal weight loss rate of 33.6%, due to the decomposition of the core material and part of the wall material of the microcapsules.

**Figure 8:**
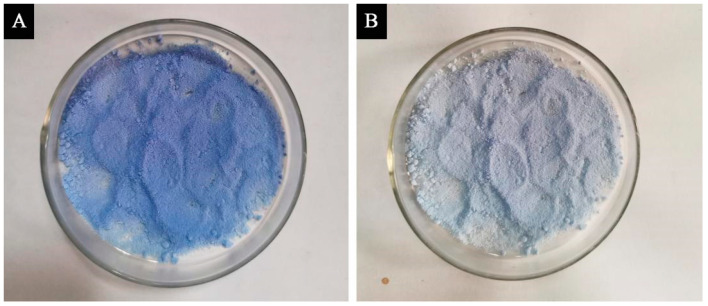
Schematic diagram of microcapsules with molar ratio of 1:1.6 at 65 °C. (**A**) before discoloration; (**B**) after discoloration.

**Figure 9:**
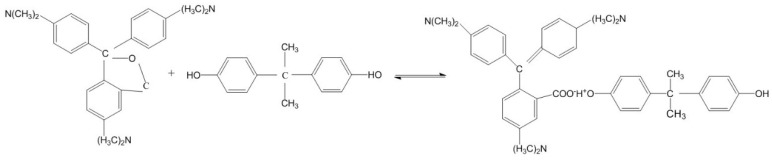
Thermochromic mechanism of microcapsules.

Figure 8 presents the schematic diagram before and after the discoloration of microcapsules with a molar ratio of urea and formaldehyde of 1:1.6. Figure 9 shows the discoloration mechanism of the thermochromic microcapsules. The crystal violet lactone was initially a closed-loop structure, and provided electronic compounds in the discoloration system, while bisphenol A was used as an electron absorbing compound. When the temperature was low, the -OH of bisphenol A reacted with the central carbon atom of crystal violet lactone; the crystal violet lactone ring broke, formed a conjugated double bond structure, and the discolored complex appeared blue. At high temperature, due to the melting of the solvent, the conjugated double bond broke, and the crystalline violet lactone reconstituted its lactone ring structure, resulting in a colorless microcapsule.

### 3.4. Effect of Microcapsule Content on the Color Difference of Paint Film

**Figure 10:**
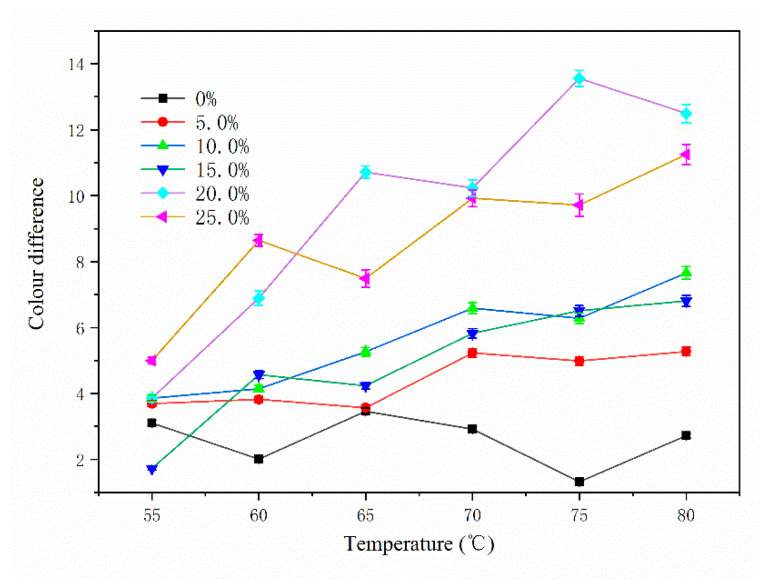
Variation with temperature of the color difference of the paint film.

**Figure 11:**
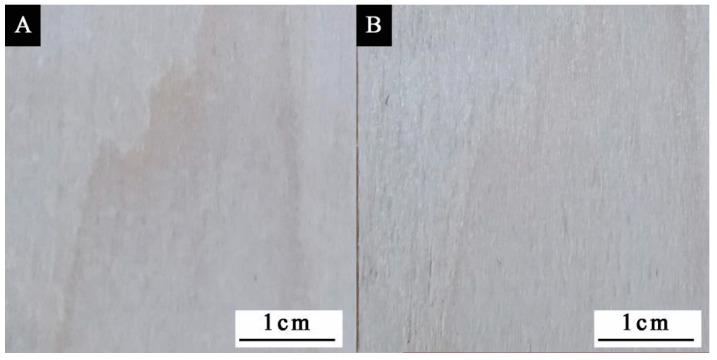
Substrate before and after thermal treatment: (**A**) before; (**B**) after.

**Figure 12:**
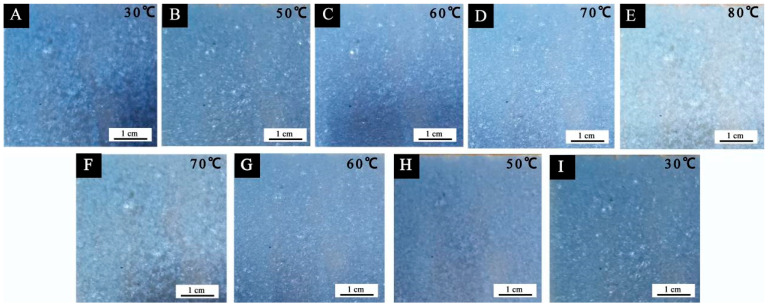
Color change of paint film at different temperatures: (**A**–**E**) temperature rise; (**F**–**I**) temperature drop.

The chromaticity values of the paint films with different microcapsule contents at different temperatures are shown in Table 14, and the change in the color difference with temperature is shown in Figure 10. It can be seen from the figure that the color difference of the paint film without microcapsules fluctuated little with the change in temperature. The color difference of the paint film with microcapsules increased gradually with increasing temperature. At about 80 °C, the color difference of the paint film was the greatest. With increasing microcapsule content, the color difference of the paint film showed an upward trend. When the microcapsule content was 0~10%, the color change of paint film surface was not obvious, and the color difference was small. The overall color difference was less than 8.0. When the microcapsule content was 20%, the color difference change of the paint film was at its most obvious. When the microcapsule content in the paint film was low, the microcapsules were not able to fully cover the surface of the substrate, and the color of the wood at room temperature changed little. Therefore, the color difference of the paint film changed little with increasing temperature. When the microcapsule content in the paint film was high, although the surface of the substrate became blue at room temperature, the color difference of the paint film did not change significantly with increasing temperature. This is because there were too many microcapsules in the paint film. During the heating process, the temperature decreased layer by layer in the conduction process, and the temperature of the inner microcapsule was much lower than that of the outside. Therefore, too many microcapsules in the paint film is not conducive to the discoloration effect of the paint film. Figure 11 presents a diagram of the substrate before and after heating. It can be seen from the figure that there was no change on the substrate surface after heating from room temperature to 80 °C. Figure 12 presents the color change of paint film at different temperatures. It can be seen from the figure that the color of the paint film gradually became lighter with increasing temperature, and the color of the paint film gradually recovered when the temperature decreased. It can be seen that the paint film with microcapsules has reversible thermochromic properties.

**Figure 13:**
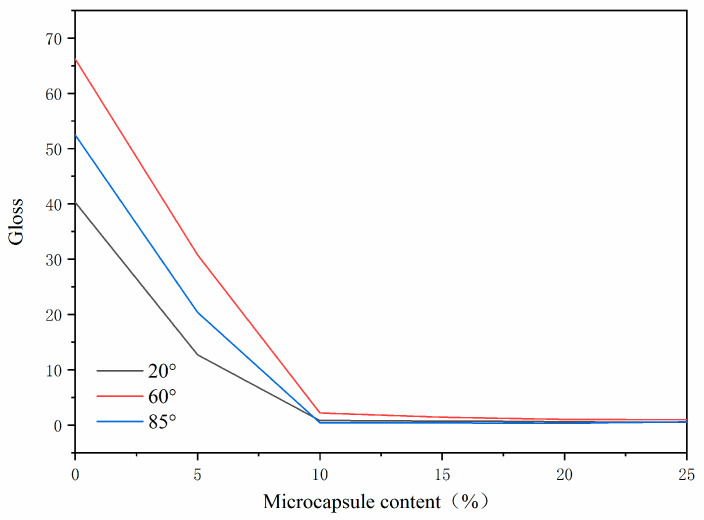
Gloss of paint film with different microcapsule content.

The effects of different microcapsule contents on the gloss of the paint film are shown in Figure 13. With the increase of microcapsule content, the gloss of the paint film decreased gradually. When the thermochromic microcapsule content was 0~10%, the gloss of the paint film decreased sharply. When the microcapsule content was 15%~25%, the gloss of the paint film changed slightly. The smoothness and roughness of the paint film surface affect the gloss of the paint film. Increasing the microcapsule content in the paint film will increase the roughness of the paint film, thus reducing the light reflection ability and gloss of the paint film.

The influence of microcapsule content on the mechanical properties of the paint film is shown in Table 15. With increasing microcapsule content, the hardness of the paint film gradually decreased. When the microcapsule content was 0~10%, the hardness of the paint film was 2H. With the microcapsule content was increased, the hardness of the paint film decreased to HB, and the decline was relatively gentle. The adhesion remained unchanged. The adhesion grade of the paint film with a content of 0~25% was grade 1. This is because the particle size of the microcapsules was small, meaning that the dispersion of the microcapsules in the paint film was better, and thus the adhesion of the paint film remained unchanged. The impact resistance first increased and then decreased. When the microcapsule content in the paint film was 20%, the impact resistance of the paint film was the best, which was 17.00 ± 0.42 kg∙cm. This is because the microcapsules were small particles with a shell–core structure [36,37]. The shell material of the microcapsules is able to protect the core material to a certain extent. When microcapsules were added to the paint film and coated on the surface of substrate, the microcapsule particles form a protective layer, which improves the impact resistance of the paint film.

**Figure 14:**
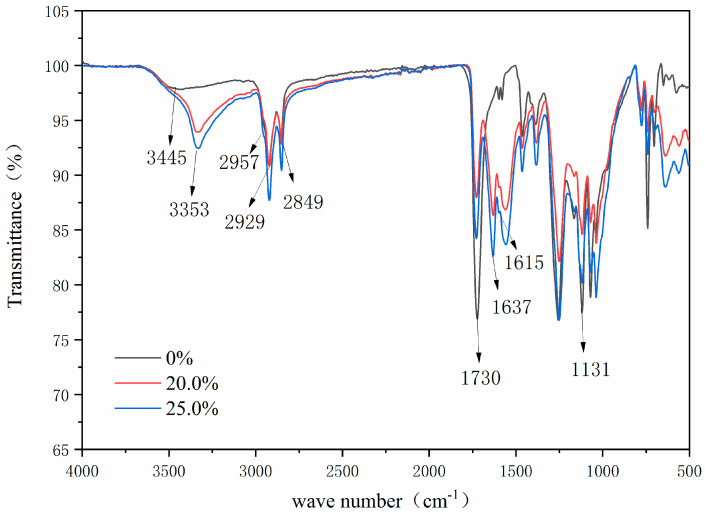
FTIR of paint film with microcapsules.

Figure 14 shows the infrared spectra of the paint film with thermochromic microcapsules and the paint film without microcapsules. For the paint film without microcapsules, the peak at 3445 cm^−^^1^ was the stretching vibration peak of the hydroxyl group, the characteristic peak of the C=O bond in ester at 1730 cm^−1^, and the characteristic peak of C-H in methyl and methylene at 2929 cm^−1^ and 2849 cm^−1^ [38]. These are the characteristic peaks of alkyd resin paint film. These characteristic peaks could also be seen in the paint film with microcapsules. In addition, there were characteristic peaks of thermochromic microcapsules. The peak at 3353 cm^−1^ was the stretching vibration peak of the N-H bond and the O-H bond, the peak at 2957 cm^−1^ was the asymmetric stretching vibration peak of CH_2_, the peak at 1637 cm^−1^ was the carbonyl stretching vibration peak of the secondary acyl, and the peak at 1131 cm^−1^ was the characteristic absorption peak of CH_3_O. These peaks indicated the successful preparation of urea formaldehyde resin. The absorption peak of ester carbonyl C=O with a non-lactone ring structure appeared at 1615 cm^−1^, and the peak at 1380 cm^−1^ corresponded to the symmetrical stretching absorption peak of carboxylate. There was no disappearance or addition of characteristic peaks, indicating that there was no chemical reaction between microcapsules and paint film.

Table 16 presents the chromaticity values of paint film with 20% thermochromic microcapsules and that without microcapsules before and after aging [39]. The paint film with microcapsules was blue before aging, and after aging, the paint film became white, with a color difference of 22.23 ± 0.55. After aging for 144 h, the paint film lost its discoloration properties. This is because the conjugated double bond structure in the core of the thermochromic microcapsule broke under the continuous irradiation of ultraviolet light, and therefore, the color of the paint film was white at room temperature. In addition, the lactone ring structure of crystalline violet lactone was also decomposed and damaged, so the paint film no longer had the discoloration capability. In the process of UV aging, the paint film on the wood surface showed obvious yellowing without microencapsulation, with a color difference of 7.26 ± 0.18. This is because the film-forming material of alkyd resin paint was sensitive to ultraviolet light, being prone to degradation and crosslinking reaction under ultraviolet radiation, resulting in irreversible yellowing of the paint film [40].

## 4. Conclusions

In this paper, thermochromic microcapsules were prepared by in situ polymerization, and the effect of the thermochromic microcapsule content of the coatings on the properties of the paint film was investigated. The orthogonal experiment found that the best preparation process was as follows: core material: wall material 4:7, emulsifier concentration 5%, and rotating speed 1600 rpm. The molar ratio of urea and formaldehyde had the greatest influence on the coating rate of microcapsules. The single-factor optimization experiment was carried out with the molar ratio of urea and formaldehyde as the variable. When the molar ratio of urea and formaldehyde was 1:1.6, the microcapsule performance was the best. At that point, the discoloration temperature of microcapsules was 65 °C, the yield was 14.58 ± 0.36 g, and the coating rate was 56.60 ± 1.41%. When microcapsules were added to the coatings, it was found that when the microcapsule content was 20%, the performance of the paint film was the best. At that point, the discoloration temperature was 80 °C, the color difference is the largest, the gloss of the paint film was 1.0, the hardness was HB, the adhesion was grade 1, and the impact resistance was 17.00 ± 0.42 kg∙cm. These results lay a foundation for the application of thermochromic coatings on furniture.

## Figures and Tables

**Table 1:** List of experimental materials.

Experimental Materials	Molecular Mass (g/mol)	CAS	Manufacturer
Crystal violet lactone	415.52	1552–42–7	Wuhan Huaxiang Biotechnology Co., Ltd., Wuhan, China
Bisphenol A	228.28	80–05–7	Wuhan Kanos Technology Co., Ltd., Wuhan, China
Tetradecanol	214.38	112–72–1	Guangzhou Jiangshun Chemical Technology Co., Ltd., Guangzhou, China
Urea	60.06	57–13–6	Guangzhou Suixin Chemical Co., Ltd., Guangzhou, China
37% formaldehyde solution	30.03	50–00–0	Jinan Huasheng Chemical Co., Ltd., Jinan, China
Triethanolamine	149.19	102–71–6	Shandong Chengkai New Material Co., Ltd., Shandong, China
Citric acid monohydrate	502.51	99026–99–0	Jinan Xiaoshi Chemical Co., Ltd., Jinan, China
Gum acacia	-	9000–01–5	Nanjing Jinyou Biotechnology Co., Ltd., Nanjing, Liaocheng, China
Absolute ethanol	46.06	64–17–5	Guangzhou Chengyi Nuoyi Instrument Co., Ltd., Guangzhou, China
Acetone	58.08	67–64–1	Guangzhou Jiangshun Chemical Technology Co., Ltd., Guangzhou, China
Ethyl acetate	88.10	141–78–6	Jinan Zhengkang Chemical Co., Ltd., Jinan, China
Xylene	106.16	1330–20–7	Suzhou Tianke Trading Co., Ltd., Suzhou, China
Acetic acid	60.05	64–19–7	Jinan Xiaoshi Chemical Co., Ltd., Jinan., China
Hydrochloric acid	36.46	7647–01–0	Jinan Xiaoshi Chemical Co., Ltd., Jinan., China

**Table 2:** Influencing factors and level of orthogonal experiment.

Level	Urea: Formaldehyde	Core Material: Wall Material	Emulsifier Concentration (%)	Speed (rpm)
1	1:1	1:7	3	1000
2	1:1.5	4:7	4	1300
3	1:2	7:7	5	1600

**Table 3:** Orthogonal experimental design of thermochromic microcapsules.

Experiment Number	Urea: Formaldehyde	Core Material: Wall Material	Emulsifier Concentration (%)	Speed (rpm)
1	1:1	1:7	3	1000
2	1:1	4:7	4	1300
3	1:1	7:7	5	1600
4	1:1.5	1:7	4	1600
5	1:1.5	4:7	5	1000
6	1:1.5	7:7	3	1300
7	1:2	1:7	5	1300
8	1:2	4:7	3	1600
9	1:2	7:7	4	1000

**Table 4:** Experimental materials used to prepare the microcapsules.

Experiment Number	Urea (g)	Formaldehyde (g)	Distilled Water (mL)	Emulsifier (g)	Discoloration Complex (g)	Sodium Chloride (g)	Silica (g)
1	3.00	4.05	90.00	0.26	0.64	0.09	0.09
2	3.00	4.05	90.00	1.37	2.57	0.36	0.36
3	3.00	4.05	90.00	3.00	4.50	0.63	0.63
4	3.00	6.08	105.00	0.40	0.75	0.10	0.10
5	3.00	6.08	105.00	2.00	3.00	0.42	0.42
6	3.00	6.08	105.00	2.10	5.25	0.74	0.74
7	3.00	8.10	120.00	0.57	0.85	0.12	0.12
8	3.00	8.10	120.00	2.28	3.40	0.48	0.48
9	3.00	8.10	120.00	3.20	6.00	0.85	0.85

**Table 5:** List of materials for single-factor experiment.

Experiment Number	Molar Ratio of Urea to Formaldehyde	Urea (g)	Formaldehyde (g)	Distilled Water (g)	Emulsifier (g)	Discoloration Complex (g)	Sodium Chloride (g)	Silica (g)
10	1:1.2	8	12.62	256.00	4.87	7.31	1.03	1.03
11	1:1.4	8	14.73	272.00	5.18	7.77	1.09	1.09
12	1:1.5	8	15.79	280.00	5.33	8.00	1.12	1.12
13	1:1.6	8	16.84	288.00	5.48	8.23	1.16	1.16
14	1:1.8	8	18.95	304.00	5.79	8.69	1.52	1.52

**Table 6:** Experimental ingredients of paint film.

Microcapsule Content (%)	Weight of Microcapsules (g)	Weight of Coatings (g)
0	0	4.00
5.0	0.20	3.80
10.0	0.40	3.60
15.0	0.60	3.40
20.0	0.80	3.20
25.0	1.00	3.00

**Table 7:** List of experimental materials.

Testing Machine	Machine Model	Manufacturer
Electronic balance	JCS-W	Harbin Zhonghui weighing instrument Co., Ltd., Harbin, China
Collector type constant temperature heating magnetic stirrer	DF−101S	Gongyi Yuhua Instrument Co., Ltd., Gongyi, China
Ultrasonic emulsifying disperser	TL−650CT	Shanghai Xinnuo Instrument Group Co., Ltd., Shanghai, China
Pear-shaped separating funnel	60ML	Nanjing kangluoda Experimental Technology Co., Ltd., Nanjing, China
Circulating water multipurpose vacuum pump	SHZ-D	Zhengzhou biochemical instrument Co., Ltd., Zhengzhou, China
Electric heating constant temperature blast drying oven	DHG−9643BS-Ⅲ	Shanghai Xinmiao medical device manufacturing Co., Ltd., Shanghai, China
Biological microscope	Zeiss Axio Scope A1	Shenzhen Guanggu Optical Instrument Co., Ltd., Shenzhen, China
Scanning electron microscope	Quanta−200	Shenzhen Sanhao Instrument Equipment Co., Ltd., Shenzhen, China
Fourier transform infrared spectrometer	VERTEX 80V	Xiamen Qunlong Instrument Co., Ltd., Xiamen, China
Colorimeter	SEGT-J	Beijing Shidai peak Technology Co., Ltd., Beijing, China
Gloss meter	DT60	Changzhou dude Precision Instrument Co., Ltd., Changzhou, China
Thermogravimetric analyzer	STA8000	PerkinElmer Inc., Waltham, MA, USA

**Table 8:** Visual analysis of orthogonal experiment.

Experiment Number	Urea: Formaldehyde	Core Material: Wall Material	Emulsifier Concentration (%)	Speed (rpm)	Coating Rate (%)
1	1:1	1:7	3	1000	30.00 ± 0.75
2	1:1	4:7	4	1300	48.00 ± 1.20
3	1:1	7:7	5	1600	52.00 ± 1.30
4	1:1.5	1:7	4	1600	24.00 ± 0.60
5	1:1.5	4:7	5	1000	54.00 ± 1.35
6	1:1.5	7:7	3	1300	53.00 ± 1.32
7	1:2	1:7	5	1300	10.00 ± 0.25
8	1:2	4:7	3	1600	8.00 ± 0.20
9	1:2	7:7	4	1000	2.00 ± 0.05
Mean1	43.333	21.333	30.333	28.667	
Mean1	43.667	36.667	24.667	37.000	
Mean1	6.667	35.667	38.667	28.000	
Range	37.000	15.334	14.000	9.000	

**Table 9:** Variance analysis of the orthogonal experiment.

Factor	Sum of Squares of Deviations	Freedom	F Ratio	F Critical Value	Significance
Molar ratio of urea to formaldehyde	2713.55	2	17.98	19.00	
Core material: wall material	441.55	2	2.92	19.00	
Emulsifier concentration	297.55	2	1.97	19.00	
Speed	150.88	2	1.00	19.00	
Error value	150.89	2			150.89

**Table 10:** Reagent resistance and acid–base resistance analysis of microcapsules.

Reagent	Dissolution		Color Change	
	Discoloration Compound	Thermochromic Microcapsule	Discoloration Compound	Thermochromic Microcapsule
Acetone	solution	insolubilization	colorless	invariant
Ethyl acetate	solution	insolubilization	colorless	invariant
Xylene	solution	insolubilization	colorless	invariant
Acetic acid	solution	insolubilization	colorless	invariant
Hydrochloric acid	solution	insolubilization	colorless	invariant
Triethanolamine	solution	insolubilization	colorless	invariant

**Table 11:** Yield of single-factor microcapsules.

Molar Ratio of Urea to Formaldehyde	Yield (g)
1:1.2	15.48 ± 0.38
1:1.4	15.91 ± 0.39
1:1.5	14.93 ± 0.37
1:1.6	14.58 ± 0.36
1:1.8	12.63 ± 0.31

**Table 12:** Coating rate of single-factor microcapsules.

Experiment Number	Molar Ratio of Urea to Formaldehyde	Coating Rate (%)
10	1:1.2	70.00 ± 1.75
11	1:1.4	63.30 ± 1.58
12	1:1.5	60.00 ± 1.50
13	1:1.6	56.60 ± 1.41
14	1:1.8	53.30 ± 1.33

**Table 13:** Thermochromic temperature of single microcapsule.

Experiment Number	Molar Ratio of Urea to Formaldehyde	Thermochromic Temperature (°C)
10	1:1.2	68.00 ± 1.70
11	1:1.4	75.00 ± 1.87
12	1:1.5	71.00 ± 1.77
13	1:1.6	65.00 ± 1.62
14	1:1.8	95.00 ± 2.37

**Table 14:** Chromaticity values of paint film at different temperatures.

Microcapsule Content (%)	Color Parameter	55 °C	60 °C	65 °C	70 °C	75 °C	80 °C
0	L	77.70 ± 1.94	64.00 ± 1.60	62.40 ± 1.56	81.10 ± 2.02	70.70 ± 1.76	62.30 ± 1.55
	a	11.20 ± 0.28	18.10 ± 0.452	18.50 ± 0.46	9.60 ± 0.24	15.50 ± 0.38	18.30 ± 0.45
	b	47.10 ± 1.17	32.80 ± 0.82	31.10 ± 0.77	43.40 ± 1.08	28.40 ± 0.71	26.90 ± 0.67
5	L	61.70 ± 1.54	59.00 ± 1.47	59.40 ± 1.48	64.00 ± 1.6	64.30 ± 1.60	64.30 ± 1.60
	a	15.20 ± 0.38	16.00 ± 0.40	17.10 ± 0.42	15.70 ± 0.39	15.30 ± 0.38	14.80 ± 0.37
	b	35.50 ± 0.88	33.20 ± 0.83	33.10 ± 0.82	38.20 ± 0.95	40.00 ± 1.00	39.70 ± 0.99
10	L	61.90 ± 1.54	59.00 ± 1.47	62.50 ± 1.56	63.70 ± 1.59	63.10 ± 1.57	63.80 ± 1.59
	a	7.90 ± 0.19	11.00 ± 0.27	10.60 ± 0.26	10.50 ± 0.26	11.70 ± 0.29	10.80 ± 0.27
	b	27.00 ± 0.67	35.70 ± 0.89	30.10 ± 0.75	31.20 ± 0.78	32.20 ± 0.80	32.20 ± 0.80
15	L	60.50 ± 1.51	59.50 ± 1.48	64.60 ± 1.61	62.90 ± 1.57	63.30 ± 1.58	61.70 ± 1.54
	a	6.80 ± 0.17	8.20 ± 0.20	7.90 ± 0.197	9.10 ± 0.22	8.40 ± 0.21	9.40 ± 0.23
	b	24.50 ± 0.61	24.90 ± 0.62	26.20 ± 0.65	27.10 ± 0.67	29.30 ± 0.73	30.30 ± 0.75
20	L	50.40 ± 1.26	68.90 ± 1.72	66.40 ± 1.66	69.00 ± 1.72	68.80 ± 1.72	69.70 ± 1.74
	a	−0.40 ± 0.01	0.10 ± 0.00	3.10 ± 0.077	1.80 ± 0.04	2.70 ± 0.067	2.20 ± 0.05
	b	16.00 ± 0.04	5.20 ± 0.13	9.50 ± 0.23	7.80 ± 0.19	10.40 ± 0.26	10.00 ± 0.25
25	L	63.00 ± 1.57	65.50 ± 1.63	67.10 ± 1.67	68.00 ± 1.70	69.00 ± 1.72	69.70 ± 1.74
	a	−1.10 ± 0.02	−1.00 ± 0.02	0.20 ± 0	1.80 ± 0.04	0.90 ± 0.02	2.70 ± 0.06
	b	0.75 ± 0.01	3.80 ± 0.09	6.20 ± 0.15	9.80 ± 0.24	8.80 ± 0.22	11.10 ± 0.27

**Table 15:** Mechanical properties of microcapsule paint films with different microcapsule contents.

Microcapsule Content (%)	Hardness	Adhesion (Grade)	Impact Resistance (kg∙cm)
0	2H	1	8.00 ± 0.20
5	2H	1	10.00 ± 0.25
10	2H	1	13.00 ± 0.32
15	H	1	15.00 ± 0.37
20	HB	1	17.00 ± 0.42
25	HB	1	15.00 ± 0.37

**Table 16:** Chromaticity value and color difference of paint film before and after aging.

Microcapsule Content (%)	Condition	L	a	b	ΔL	Δa	Δb	Color Difference
0	Before aging	77.55 ± 1.93	14.65 ± 0.36	37.9 ± 0.94	6.88 ± 0.17	2.18 ± 0.05	0.83 ± 0.02	7.26 ± 0.18
	After aging	70.68 ± 1.76	12.48 ± 0.31	37.08 ± 0.92				
20.0	Before aging	65.43 ± 1.63	−0.4 ± 0.01	1.60 ± 0.04	−5.00 ± 0.12	−6.12 ± 0.15	−20.77 ± 0.51	22.23 ± 0.55
	After aging	70.47 ± 1.76	5.7 ± 0.14	22.37 ± 0.55				

## Data Availability

Not applicable.
